# Acceptance and associated factors of HIV testing among college students in China: A systematic review and meta-analysis

**DOI:** 10.1371/journal.pone.0284865

**Published:** 2023-04-27

**Authors:** Shiqin Liao, Jie Li, Mingting Liu, Hongmei Xie, Yutong Lu, Yunlan Jiang

**Affiliations:** 1 School of Nursing, Chengdu University of Traditional Chinese Medicine, Chengdu, Sichuan Province, China; 2 Nursing Department, Hospital of Chengdu University of Traditional Chinese Medicine, Chengdu, Sichuan Province, China; University of Buea, CAMEROON

## Abstract

**Background:**

Although HIV testing is helpful for early detection and treatment of HIV, its utilization rate is low among college students in China. Understanding the acceptance and associated factors of HIV testing is the key to improve the detection rate. The purpose of the systematic review was to examine the acceptance and associated factors of HIV testing (including HIV self-testing and HIV counseling and testing services) among college students in China.

**Methods:**

This systematic review was reported following PRISMA guidelines 2020. Electronic sources such as PubMed, Embase, Web of Science, CNKI, CBM, Wanfang Database and VIP Database were searched for relevant studies published before September 2022. The tool by Agency for Healthcare Research and Quality (AHRQ) was used to assess quality for cross-sectional studies. The random-effects and fixed-effect model were employed to estimate the pooled proportions and associated factor of HIV testing acceptance. The Cochrane’s Q statistic and I^2^ test were used to examine heterogeneity. All the quantitative meta analyses were conducted using STATA version 12 software.

**Results:**

A total of 21 eligible studies with 100, 821 participants were included in the systematic review. The pooled acceptance rate of HIV testing was 68% (95% CI = 60, 76), and varies between regions in China. Male, heterosexual and urban college students had higher HIV testing acceptance. Gender, medical specialty, sexual education, sexual behavior, HIV/AIDS knowledge, perception HIV risk, and previous HIV testing were the factors associated with HIV testing acceptance.

**Conclusion:**

The review revealed that most of the college students intend to accept HIV detection, and the proportion of acceptance influenced by different factors. Therefore, the government and universities should implement targeted measures, improve HIV testing services, and promote HIV testing behavior.

**Systematic review registration:**

PROSPERO CRD42022367976.

## Introduction

Despite substantial advances in understanding and treating HIV in the past decades, it remains a formidable challenge of the global public health [1, 2]. In recent year, the overall HIV incidence in Chinese population has declined, but the infection rate among young students has increased [3]. The cases of HIV infection among young students had been estimated more than 140,000 from 2010 to 2019 in China [4]. According to the Chinese Center for Disease Control and Prevention, the growth rate of new HIV infection among college students has reached to 30%-50% per year [5]. Moreover, sexual contact is the main approach of HIV transmission among young students [6]. Chinese college students have a higher risk of HIV infection due to the liberalization of sexual concepts, lack of sexual health education, and gradually increasing unprotected sexual behaviors [7]. Therefore, college students have been recognized a priority population for HIV prevention and control in our country.

95% of people living with HIV aware their infection status is the primary step in achieving the Joint United Nations Program on HIV and AIDS (UNAIDS) 95-95-95 targets by 2030 [8]. The UNAIDS targets have not been reached in China despite several efforts, and only 75.7% of individuals living with HIV are aware of their status [9]. HIV testing is helpful for infected people to discover their status earlier, which is crucial for seeking antiviral treatment and preventing the spread of HIV [10, 11]. Worryingly, the prevalence of HIV testing among Chinese college students is lower than national average. Only 44.3% of young male students with MSM have HIV testing in our country [12]. In an effort to popularize early detection of HIV and to control the HIV epidemic, the government gradually increases HIV testing services among universities. For example, a free HIV counseling and testing (HCT) and an anonymous urine self-collection for HIV self-testing (HIVST) are provided on college campuses [13, 14]. Unfortunately, due to the lack of HIV testing knowledge, discriminatory attitudes and HIV-related stigma, the utilization rate of these services are low of Chinese college students [15, 16].

The acceptance of HIV testing is one of the important elements influencing testing behavior, and their relationship is positively correlated [17]. Clarifying the willingness for HIV testing is critical to adopt strategies to improve the recognition of HIV testing among college students, as well as to promote active detection. Furthermore, the surveys on HIV testing acceptance are conducive to identify college students’ awareness of detection and weak points in practice, so as to implement interventions precisely. While there are many relevant studies on the acceptance of HIV testing among college students in China, the results are inconsistent and quantitative analysis has not been performed yet. With the promotion and application of multiple new HIV testing methods, the acceptability of HIV testing will be affected [18]. Moreover, it is necessary to understand factors that are associated with HIV testing willingness. Therefore, the objective of this meta-analysis is to examine the prevalence and associated factors of HIV testing willingness among college students in China. The finding generated from this review will provide scientific evidence to assist healthcare department and researchers to formulate policies and guidelines for improving the acceptance and behavior of HIV testing in college students.

## Materials and methods

### Protocol and registration

This systematic review was reported in accordance with the ‘Preferred Reporting Items for Systematic Reviews and Meta-analyses’ (PRISMA) [19] (S1 File). The protocol of the systematic review was registered with PROSPERO (registration number CRD42022367976).

### Eligibility criteria

Studies were considered if they: reported the acceptance, risk and protective factors, and disparities among college students associated with HIV testing (including HIVST and HCT) in China; were published in English or Chinese; provided the prevalence and odds ratios (OR) with 95% confidence intervals (95% CI), or enough data to calculated them; were conducted in China. However, reviews, conference literature, and studies in which data could not be transformed were excluded.

### Search strategy

A systematic search of PubMed, Web of science, Embase, the China National Knowledge Infrastructure (CNKI), Chinese Biomedical Literature Database (CBM), Wanfang Database, and VIP Database was undertaken on September 30, 2022. Medical Subject Heading (MeSH) terms and relevant text words including “HIV testing” OR “AIDS testing” OR “HIV diagnosis” OR “HIV counseling and testing” OR “HCT” AND “willingness” OR “attitude*” OR “accept*” AND “college students” OR “university students” OR “undergraduate” AND “China” OR “Chinese” were searched in the above databases. In order to prevent omissions, we checked the reference lists of included articles and relevant reviews. The full search strategy for PubMed is obtained in S2 File.

### Study selection

All searched articles were uploaded into the EndNote X9 citation manager. After removing duplicate articles, two researchers (LSQ and LJ) independently screened all the titles and abstracts according to the criteria. After the titles and abstracts screening, the remain records were assessed by reading full-text to confirm final results. Any discrepancies in selection were resolved through discussion with the third researchers (JYL).

### Data extraction

Two researchers (LSQ and LJ) extracted data from included studies simultaneously and cross-checked for inconsistencies. Data extraction was performed using standardized form in the Microsoft Excel. The following information was collected: first author, year of publication, investigation time, sample size, study design, region of study, prevalence of HIV testing acceptance and associated factors with HIV testing acceptance (OR with 95% CI). We contacted the author of the original articles for further information when necessary.

### Quality assessment

The assessment tool designed by the Agency for Healthcare Research and Quality (AHRQ) for cross-sectional research was used to evaluate the methodological quality of each included studies [20]. The tool’s 11 items were evaluated, and research that met one of them received one point. Moreover, the study scored of 8–11 was classified as “high quality”, 4–7 as “moderate quality”, and 0–3 as “low quality”. The quality of the eligible studies was appraised by two independent reviews (LSQ and LJ) and verified by the third reviewers (JYL).

### Data synthesis

All the quantitative statistical analyses were conducted using STATA Version 12 software. The proportion of HIV testing acceptance was estimated with standard error as effect measure, and presented using a forest plot. Besides, the associated factors of testing willingness were estimated with OR and its 95% CI when these included at least two studied. Heterogeneity was assessed using Cochrane’s Q statistic and I^2^ test, and I^2^ > 75% defined as high heterogeneity [21]. A random-effects model analysis was used to pool the results with the I^2^ ≧ 50%, conversely, a fixed-effects model analysis was used. Subgroup analyses were performed based on survey time, gender, residence, region of study, sexual orientation and testing approach. The robustness of the result was assessed by a leave-one-out sensitivity analysis. To investigate the potential publication bias, Egger’s regression test was employed (P < 0.05 indicated that publication bias exists) [22].

## Results

### Search outcome

**Fig 1:**
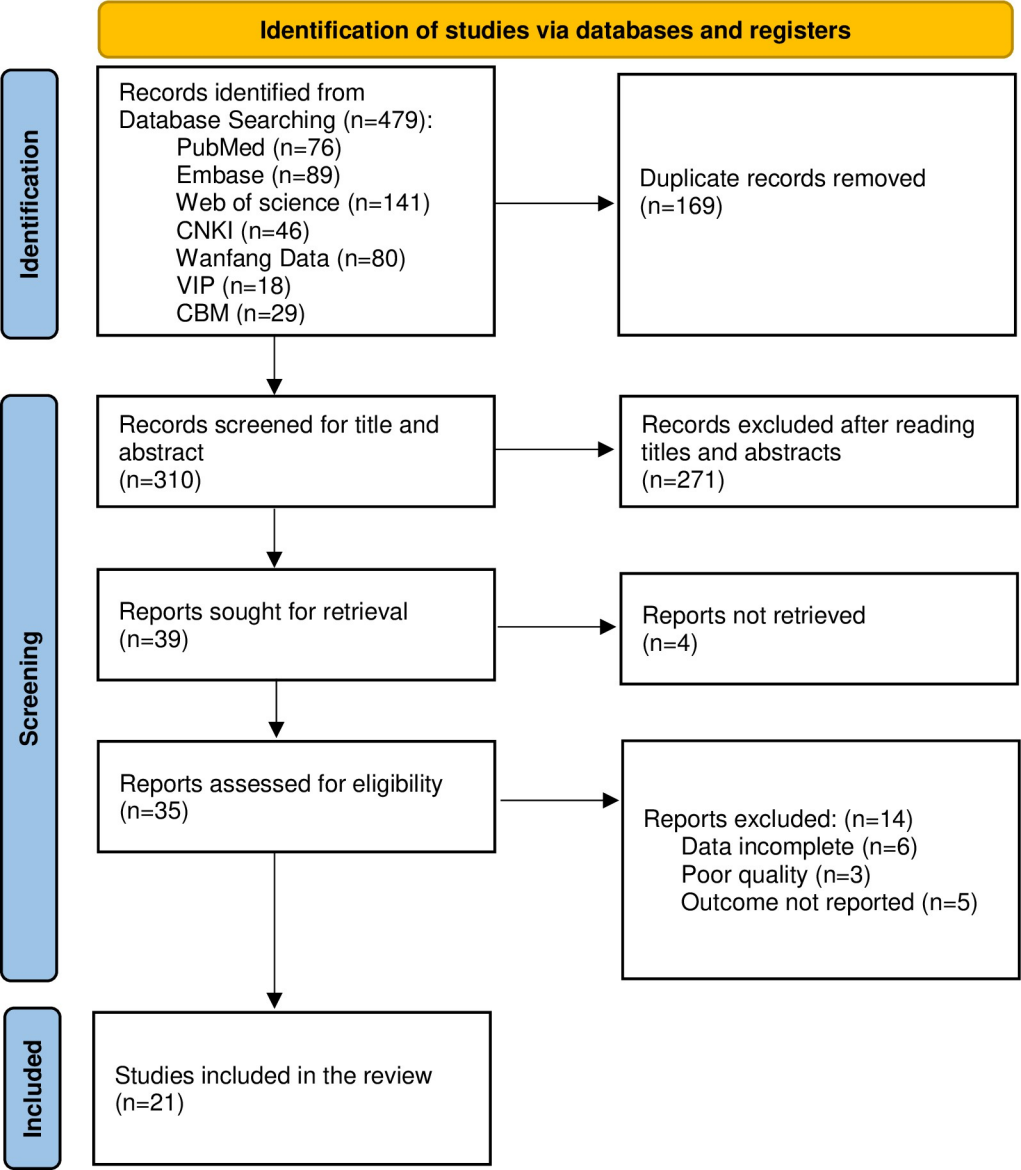
PSIAMA flow diagram of study selection.

After removal of duplicates, a total of 310 records were identified through electronic databases. Of these, 275 records were discarded by screening the title and abstract and an additional 14 records were excluded after full-text review. Finally, 21 studies were retained for the meta-analysis, involving 100,821participants [23–43]. A PRISMA flow diagram of study selection provided in Fig 1.

### Characteristics of included studies

A total of 21 studies with sample sizes ranging from 353 to 60,849 living in China were included in the study. The included cross-sectional studies were respectively conducted in thirteen provinces, and most used online questionnaires to collected information. Nine studies reported the willingness to HIVST [23, 24, 26, 28, 29, 37, 39, 41, 42], and six addressed the willingness to HCT [25, 27, 30–32, 43]. HIV/AIDS knowledge was the associated factors reported most frequently for the included studies, and medical specialty, gender, sexual behavior were followed. Table 1 presented more details of the studies.

**Table 1:** Characteristics of included studies.

Study and year	Region	Sample (male %)	Survey time	Age range/ mean(SD)	Testing approach	Acceptance rate (%)	Main associated factors	Quality of assessment
Bao R, 2019	Liaoning	1208 (34.35%)	Dec, 2018	18.47 (1.27)	HIVST	69.40%	Sexual behavior, sexual education, previous HIV testing	Moderate
Bi C, 2022	Shanghai	519 (28.32%)	Jul-Oct, 2018	≧ 18	HIVST	90.37%	HIV/AIDS knowledge, major, price of HIVST	High
Gao H, 2010	Tianjin	1674 (48.51%)	Not stated	21.13 (2.71)	HCT	78.70%	Attitude for HIV, age	Moderate
Guo Y, 2021	Henan	215 (40.93%)	Jun-Sept, 2019	19.79 (1.18)	HIVST	86.51%	Not stated	Moderate
Huang L, 2012	Tianjin	986 (17.44%)	Not stated	21.93 (1.30)	HCT	82.76%	HIV/AIDS knowledge, HIV discrimination	Moderate
Huang Y, 2021	Sichuan	1537 (42.03%)	Sept-Nov, 2019	18.98 (1.10)	HIVST	62.46%	Art major, sexual behavior, HIV/AIDS knowledge, HIV risk perception	Moderate
Liang M, 2022	Hunan	801 (23.97%)	Aug-Oct, 2022	19.6 (1.8)	HIVST	72.50%	Not stated	Moderate
Lin Z, 2017	Fujian	2587 (49.32%)	Oct, 2015	Not stated	HCT	89.30%	Not stated	Moderate
Liu C, 2020	Henan	2011 (45.40%)	Sept-Nov, 2019	≧ 15	HCT	59.42%	Medical specialty, sexual behavior	Moderate
Ma H, 2020	Hubei	1615 (45.51%)	Oct-Dec, 2017	18.12 (1.18)	HCT	54.70%	Gender	Moderate
Pei R, 2021	Sichuan	4133 (38.33%)	Oct-Dec, 2020	Not stated	Not stated	51.20%	Not stated	Moderate
Qin Q, 2017	Anhui	2928 (49.56%)	Nov-Dec, 2016	18.95 (1.08)	Not stated	37.60%	Not stated	Moderate
Su J, 2022	Beijing	2069 (33.69%)	Oct, 2020	20.33 (1.27)	Not stated	82.80%	HIV/AIDS knowledge, homosexuality	Moderate
Xiao D, 2021	Beijing	1248 (43.51%)	Sept, 2018	18.36 (1.00)	Not stated	62.98%	HIV/AIDS knowledge	Moderate
Yan L, 2020	Hunan	1649 (46.21%)	Jul-Nov, 2019	19.55 (0.74)	HIVST	73.30%	Medical specialty, HIV/AIDS knowledge	Moderate
Yu B,2021	Sichuan	5294 (34.15%)	Sept, 2018	19.56 (1.73)	Not stated	17.30%	Gender, medical specialty	Moderate
Zhang G, 2022	Guangxi	4264 (38.13%)	Mar-Dec, 2019	19.14 (0.58)	HIVST	57.01%	Gender, medical specialty, sexual behavior, HIV/AIDS knowledge, sexual education	High
Zhang J, 2021	Beijing	1567 (56.80%)	May-Jun, 2019	19.15 (4.22)	Not stated	55.14%	HIV/AIDS knowledge, previous HIV testing	Moderate
Zhao D, 2020	Heilongjiang	60849 (40.86%)	Dec 2017-Jan 2018	19.6 (1.6)	HIVST	73.30%	Not applicable to condoms	High
Tang Z, 2020	Yunnan	353 (49.58%)	Not stated	Not stated	HIVST	76.77%	Gender, detection accuracy	Moderate
Fu G, 2018	Hubei	3314 (33.13%)	Sept-Dec, 2016	15–26	HCT	77.90%	HIV risk perception, HIV stigma	Moderate

### Quality assessment

Three studies obtained high quality [24, 39, 41] while the remains were moderate quality [23, 25–38, 40, 42, 43] according to the AHRQ. None of the studies scored in item 5 and 11, which were related to the concealment of participant status and the follow-up data. The score of study were presented in S3 File.

### Acceptance of HIV testing in Chinese college students

**Fig 2:**
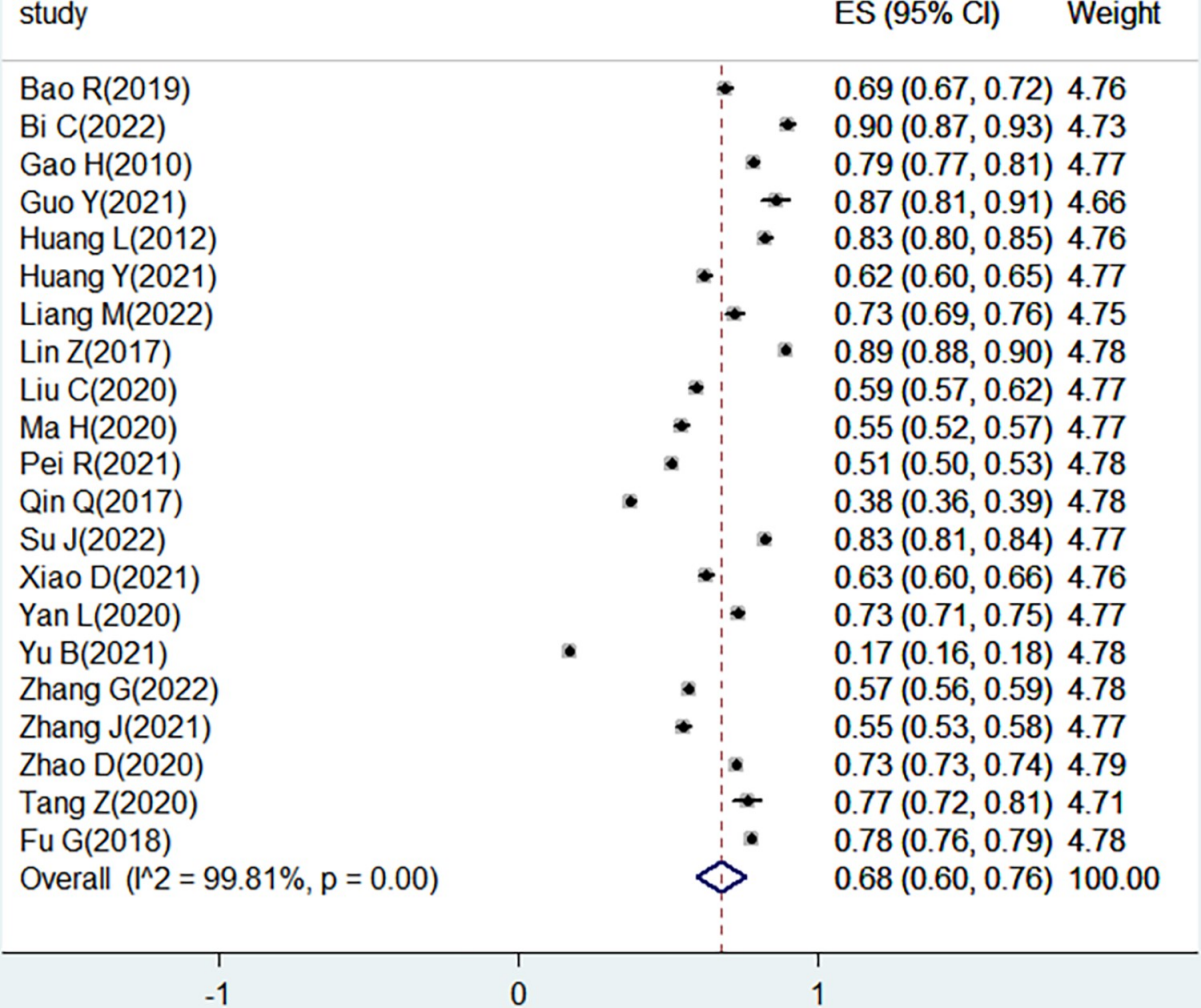
The pooled acceptance of HIV testing in Chinese college students.

A total of 21 studies reported the acceptance of HIV testing in Chinese college students. The pooled proportion of HIV testing acceptance among Chinese college students was 68% (95% CI = 60, 76). Due to the high level of heterogeneity (I^2^ = 99.81%, P < 0.001), the random-effect model was used for the analysis. A forest plot of the pooled effect size presented in Fig 2.

### Subgroup analysis

Subgroup analysis was performed based on survey time, gender, residence, region of study, sexual orientation and testing approach. The results revealed that the higher acceptance was observed among studies done in Eastern China, male students, urban students, and heterosexual students. In subgroup analysis of survey time and testing approach, the pooled prevalence of HIV testing acceptance was similar. (Table 2)

**Table 2:** Subgroup analysis on the acceptance of HIV testing in Chinese college students.

Subgroup	No. of studies	Proportion of acceptance (%) (95%CI)	I^2^	p -value
**Survey year**				
2015–2018	9	65% (47, 80)	99.91%	< 0.001
2019–2022	9	67% (59, 78)	99.15%	< 0.001
**Region**				
Eastern	9	75% (65, 84)	99.48%	< 0.001
Central	8	67% (58, 77)	99.64%	< 0.001
Western	4	52% (26, 77)	99.85%	< 0.001
**Testing approach**				
HIVST	9	74% (68, 79)	98.84%	< 0.001
HCT	6	75% (63, 85)	99.48%	< 0.001
**Gender**				
Male	11	64% (52, 75)	99.62%	< 0.001
Female	11	60% (46, 74)	99.81%	< 0.001
**Residence**				
Urban	3	59% (36, 80)	99.17%	< 0.001
Rural	4	41% (19, 65)	99.67%	< 0.001
**Sexual Orientation**				
Heterosexual	8	67% (50, 82)	99.90%	< 0.001
Non-heterosexual	8	64% (44, 82)	99.26%	< 0.001

### Associated factors of HIV testing acceptance in Chinese college students

After reviewing the associated factors of HIV testing acceptance, seven determinants were explored frequently. As shown in Table 3, male students (OR = 1.37; 95% CI: 1.25, 1.50) and medical students (OR = 1.61; 95% CI: 1.23, 2.10) increased the acceptance of HIV testing in China, respectively. In addition, students who had sexual education (OR = 1.42; 95% CI: 1.27, 1.58), experienced the sexual behavior (OR = 1.63; 95% CI: 1.16, 2.29), had knowledge about HIV/AIDS (OR = 1.75; 95% CI: 1.40, 2.19), and perceived the risk of HIV infection (OR = 1.63; 95% CI: 1.42, 1.88) were more likely to test HIV. For experience with the detection, students who had been tested associated with a significant acceptance of HIV testing (OR = 9.87; 95% CI: 2.80, 34.80).

**Table 3:** Associated factors of HIV testing acceptance in Chinese college students.

Associated factor	No. of studies	Pooled OR (95%CI)	I^2^	p -value
Male	5	1.37 (1.25, 1.50)	0%	0.766
Medical specialty	5	1.61 (1.23, 2.10)	82.6%	<0.001
Having sexual education	2	1.42 (1.27, 1.58)	0%	0.621
Having sexual behavior	4	1.63 (1.16, 2.29)	73.4%	0.010
HIV/AIDS knowledge	9	1.75 (1.40, 2.19)	79.5%	<0.001
Having HIV risk perception	2	1.63 (1.42, 1.88)	0%	0.945
Having previous HIV testing	2	9.87 (2.80, 34.80)	0%	0.962

### Sensitivity analysis and publication bias

**Fig 3:**
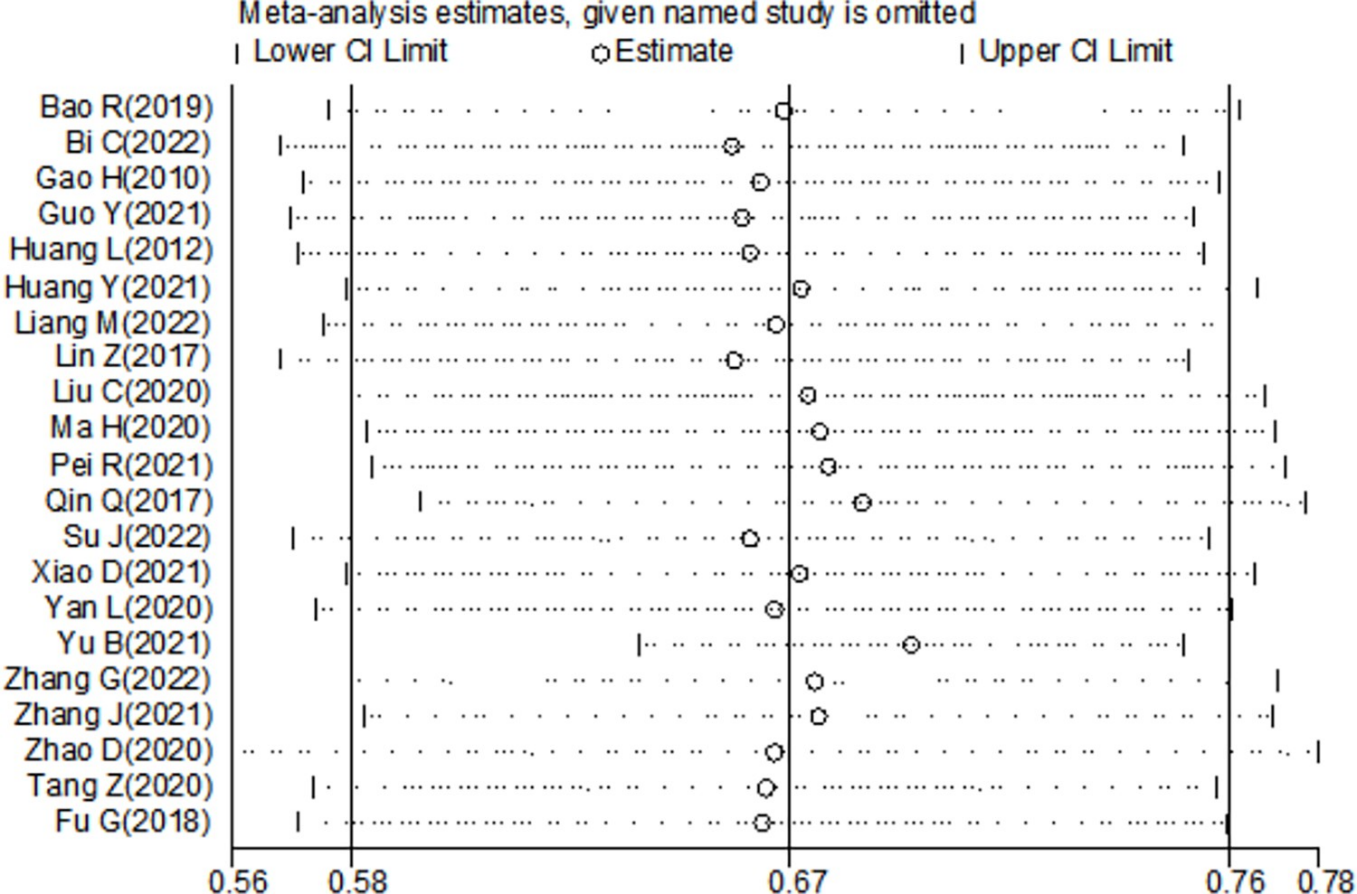
Sensitivity analysis of the 21 studies.

**Fig 4:**
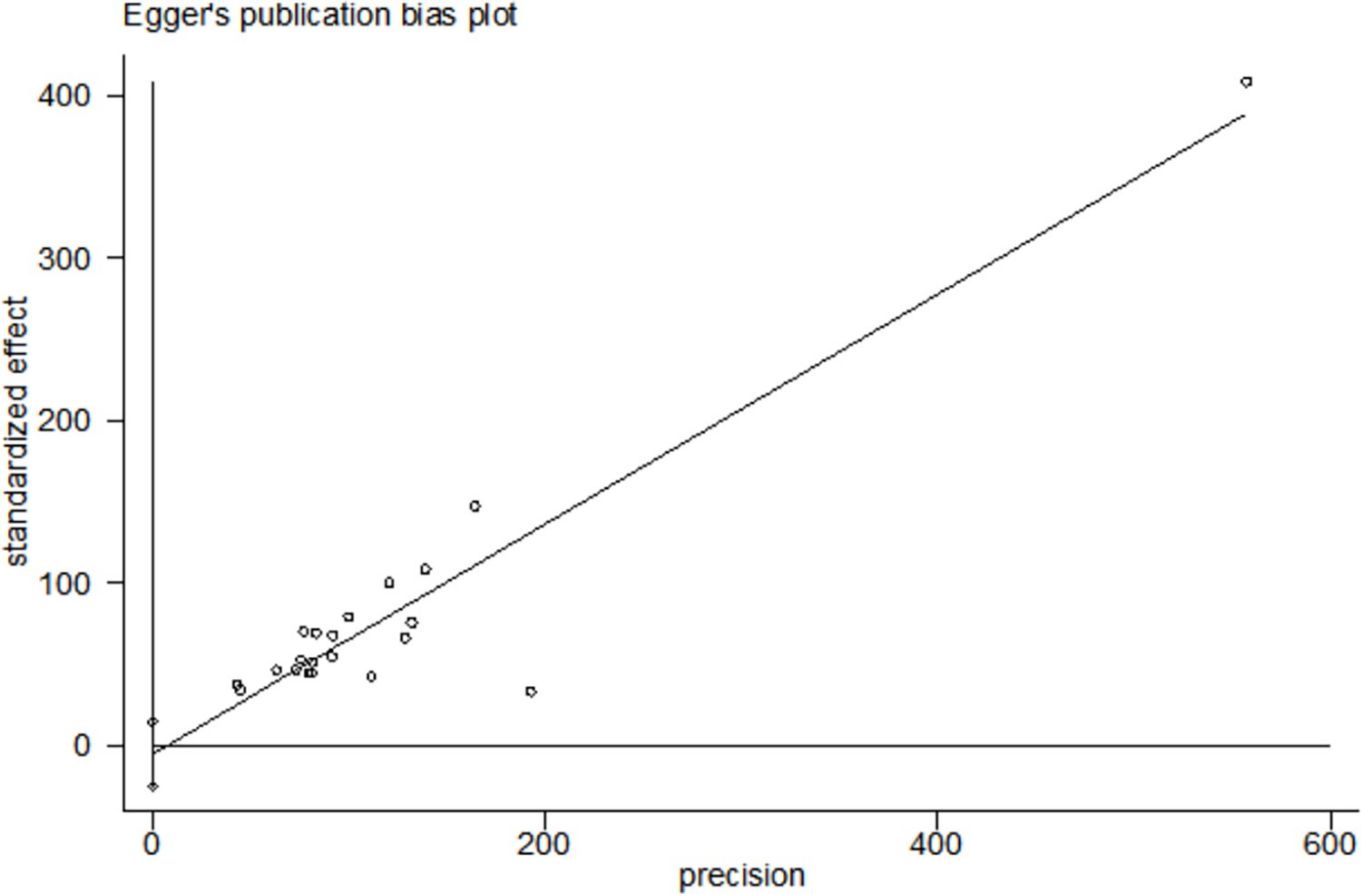
Egger’s publication bias plot of the 21 studies.

We conducted sensitivity analysis of all included studies by gradually excluding each study (Fig 3). The results were not influenced significantly with the exclusion of the study, suggesting that the stability of the result. We not found obvious publication bias of 21 studies with Egger’s regression test (P = 0.554). (Fig 4).

## Discussion

Global public health system is facing a huge challenge of in HIV prophylaxis and control. The proactive detection of HIV is critical to cut off the transmission and end the pandemic [44]. However, young students are less likely to uptake HIV testing, though they have a greater risk of exposure [45]. Investigation on the acceptance and related factors for HIV testing among college students would provide favorable evidence for government to formulate effective policies to expand the scope of testing. In this systematic review, we evaluated the college students’ acceptance rate and associated factors for HIV testing in China, indicating the importance of health management awareness and related knowledge education.

Overall, this review showed that the acceptance rate of HIV testing was 68% in Chinese college students. The pooled acceptance was slightly higher than the result revealing by an investigation in Tanzania (62.7%) [46], and lower than the proportion of willingness among adults in the United States (77.4%) [47]. The low intention could relate to the concerns about privacy exposure and unawareness of rapid detection [48]. The results of subgroup analysis suggested that the college students’ detection willingness did vary by regions and residence. In eastern China (75%) and urban areas (59%), the college students appeared to have substantially higher proportion of detection willingness. The reasons for these differences may be related to the convenience of detection services and higher level of education in economically developed places. Intriguingly, college students had a positive attitude toward HIVST (74%) and HCT (75%) service, and the rate was higher than the results conducted in Nigeria [49]. Due to the advantage of rapid, privacy, convenience, and confidentiality, HIVST had been an alternative supplementary detection method in college students [50]. However, HIV testing has not been included in the routine physical examination of college students in China, and the costs of testing method is a problem for the acceptance [51]. Notably, heterosexual students (67%) has higher acceptance of HIV testing than non-heterosexual students (64%) in our study. The difference might be caused by the shame psychology and the fear of receiving a positive outcome [52]. To improve the acceptance rate of HIV testing, health institutions should assure of confidential screening to dispel the concern about privacy exposure.

In addition, the most documented influencing factors for testing acceptance could be reduced to three aspects, including demographic characteristics, personal views, and HIV testing behavior. For demographic characteristics, male students tended to be more willing to accept HIV testing than female, which was inconsistent with the finding conducted in South Africa [53]. It might be related to the diversity of values in young people, and the gender difference would be influenced by other social behavior [54]. In our study, the finding might be explained by the high incidence of sexual behavior and unsafe sexual behavior among male students, and male students have a higher risk perception and behavior initiative [55, 56]. Secondly, the medical students were related to higher willingness to HIV testing, which was similar to the other study [15]. Medical students have more opportunities to receive medical-related information, thereby developing stronger awareness of HIV prevention and treatment. In terms of personal views, sexual behavior and sexual education were influencing factors of the acceptance for detection in college students [52, 57]. Those students have more demand for HIV testing, hence HIV screening on college campus is essential. Moreover, HIV/AIDS knowledge would increase the acceptance for detection in college students. Similar finding was also reported by Yuan L et al, recommending that education departments should explore appropriate publicity channels to eliminate discrimination and fear of HIV infection among college students, as well as expand the relevant knowledge to improve the college students’ acceptance [58]. Furthermore, the results showed that HIV risk perception was a factor associated with acceptance to be tested, and this finding was accordant with other papers [59]. Notably, most college students who refusing detection believe it’s impossible for them to be infected with HIV, and they lack the understanding of the current status for HIV infection [60]. In terms of HIV testing behavior, college students with previous HIV testing intended to seek HIV testing services actively, which was consistent with that reported by Bhalakia AM et al. [57] It may be related that they are more vulnerable to infection than other students [61]. On the one hand, it is necessary to disseminate HIV-related knowledge and mitigate the negative impact of rumors to eliminate detection hesitancy among college students. On the other hand, the government should constantly popularize the innovative methods for HIV testing and broaden areas of detection.

## Strengths and limitations

The review includes some positive aspects. First, the included studies were considered primarily evidence of moderate and high levels, and covered the majority of regions in China, making them fairly representative. Second, this review explored some elements that may promote the acceptance of HIV testing, and most of this element were adjusted with potential confounding factors. Third, to our knowledge, this study is the first systematic review and meta-analysis of acceptance for HIV testing in China. However, some limitations are included in this study. Considering a significant heterogeneity of the pooled proportions, we performed the random-effect model, sensitivity analysis and subgroup analysis to identify the sources of heterogeneity. Unfortunately, we did not find an obvious reduction of heterogeneity. Bases on these, we consider that the difference with sample size, questionnaire details might affect the heterogeneity. In addition, the cross-sectional design of all the collected literature may have limitations for our review. Finally, the number of articles included in the review is limited, which might make a deviation in inference.

## Conclusion

In conclusion, the acceptance proportion of HIV testing (especially the HIVST and HCT) was high among Chinese college students, and varied with different characteristics. Male and medical students were more likely to accept detection. Sex education, sexual behavior, knowledge of HIV/AIDS, HIV risk perception, and previous HIV testing were important associated factors in students’ willingness. Combined with the situation of low HIV testing, the healthcare department should convey trusted information, provide convenient testing services, as well as develop intervention measures based on the above determinants to promoting college students to seek HIV testing.

## Supporting information

S1 File PRISMA 2020 checklist. (PDF)

S2 File Search strategy for PubMed. (PDF)

S3 File Quality assessment of included studies by AHRQ. (PDF)
