# Supplementary material for: Acceptance and associated factors of HIV testing among college students in China: A systematic review and meta-analysis
Source: PLoS One. 2023 Apr 27;18(4):e0284865. doi: 10.1371/journal.pone.0284865 (PMC10139193; doi:10.1371/journal.pone.0284865)
Supplement: S2 File — (PDF) [file pone.0284865.s002.pdf]

Search strategy for PubMed

#1 HIV testing[Mesh]

#2 HIV testing[Title/Abstract] OR AIDS testing[Title/Abstract] OR HIV diagnosis[Title/Abstract] OR HIV counseling and testing[Title/Abstract] OR HCT[Title/Abstract]

#3 willingness[Title/Abstract] OR attitude*[Title/Abstract] OR accept*[Title/Abstract]

#4 college students[Title/Abstract] OR university students[Title/Abstract] OR undergraduate [Title/Abstract]

#5 China[Title/Abstract] OR Chinese[Title/Abstract]

#6 #1 OR #2

#7 #6 AND #3 AND #4 AND #5
