# Supplementary material for: Acceptance and associated factors of HIV testing among college students in China: A systematic review and meta-analysis
Source: PLoS One. 2023 Apr 27;18(4):e0284865. doi: 10.1371/journal.pone.0284865 (PMC10139193; doi:10.1371/journal.pone.0284865)
Supplement: S3 File — (PDF) [file pone.0284865.s003.pdf]

ARHQ checklist for cross-sectional study

1. Define the source of information (survey, record review).
2. List inclusion and exclusion criteria for exposed and unexposed subjects (cases and controls) or refer to previous publications.
3. Indicate time period used for identifying patients.
4. Indicate whether or not subjects were consecutive if not population-based.
5. Indicate if evaluators of subjective components of study were masked to other aspects of the status of the participants.
6. Describe any assessments undertaken for quality assurance purpose (e,g, test/retest of primary out come measurements).
7. Explain any patient exclusions from analysis.
8. Describe how confounding was assessed and/or controlled.
9. If applicable, explain how missing data were handled in the analysis.
10. Summarize patient response rates and completeness of data collection.
11. Clarify what follow-up, if any, was expected and the percentage of patients for which incomplete data or follow-up was obtained.

Quality assessment(Y-Yes, N-No, U-Unclear)

**Study**	**1**	**2**	**3**	**4**	**5**	**6**	**7**	**8**	**9**	**10**	**11**	**Total score**
Bao R	Y	Y	Y	Y	U	Y	N	Y	N	Y	N	7
Bi C	Y	Y	Y	Y	U	Y	Y	Y	N	Y	N	8
Gao H	Y	Y	N	Y	U	N	N	Y	N	Y	N	5
Guo Y	Y	Y	Y	Y	U	Y	N	N	N	Y	N	6
Huang L	Y	Y	N	Y	U	Y	N	Y	N	Y	N	6
Huang Y	Y	Y	Y	Y	U	Y	N	Y	N	Y	N	7
Liang M	Y	Y	Y	Y	U	Y	N	N	N	Y	N	6
Lin Z	Y	Y	Y	Y	U	N	N	N	N	Y	N	5
Liu C	Y	Y	Y	Y	U	N	N	Y	N	Y	N	6
Ma H	Y	Y	Y	Y	U	Y	N	Y	N	Y	N	7
Pei R	Y	Y	Y	Y	U	Y	N	N	N	Y	N	6
Qin Q	Y	Y	Y	Y	U	Y	N	N	N	Y	N	6
Su J	Y	Y	Y	Y	U	Y	N	Y	N	Y	N	7
Xiao D	Y	Y	Y	Y	U	Y	N	Y	N	Y	N	7
Yan L	Y	Y	Y	Y	U	Y	N	Y	N	Y	N	7
Yu B	Y	Y	Y	Y	U	Y	N	Y	N	Y	N	7
Zhang G	Y	Y	Y	Y	U	Y	Y	Y	N	Y	N	8
Zhang J	Y	Y	Y	Y	U	Y	N	Y	N	Y	N	7
Zhao D	Y	Y	Y	Y	U	Y	Y	Y	Y	Y	N	9
Tang Z	Y	Y	N	Y	U	N	N	Y	N	Y	N	5
Fu G	Y	Y	Y	Y	U	Y	N	Y	N	Y	N	7
